# Challenges and opportunities of integration of community based Management of Acute Malnutrition into the government health system in Bangladesh: a qualitative study

**DOI:** 10.1186/s12913-018-3087-9

**Published:** 2018-04-10

**Authors:** Santhia Ireen, Mohammad Jyoti Raihan, Nuzhat Choudhury, M. Munirul Islam, Md Iqbal Hossain, Ziaul Islam, S. M. Mustafizur Rahman, Tahmeed Ahmed

**Affiliations:** 1 Nutrition and Clinical Services Division, International Centre for Diarrhoeal Disease Research, Bangladesh, Dhaka, Bangladesh; 2 Health Systems and Population Studies Division, International Centre for Diarrhoeal Disease Research, Dhaka, Bangladesh; 3 Institute of Public Health Nutrition and National Nutrition Services, Ministry of Health and Family Welfare, Government of the People’s Republic of Bangladesh, Dhaka, Bangladesh

**Keywords:** Bangladesh, Community based management of acute malnutrition, Health systems, Ready-to-use therapeutic foods, Severe acute malnutrition

## Abstract

**Background:**

Severe acute malnutrition (SAM) in children is the most serious form of malnutrition and is associated with very high rates of morbidity and mortality. For sustainable SAM management, United Nations recommends integration of community based management of acute malnutrition (CMAM) into the health system. The objective of the study was to assess the preparedness of the health system to implement CMAM in Bangladesh.

**Methods:**

The assessment was undertaken during January to May 2014 by conducting document review, key informant interviews, and direct observation. A total of 38 key informant interviews were conducted among government policy makers and program managers (*n* = 4), nutrition experts (*n* = 2), health and nutrition implementing partners (*n* = 2), development partner (*n* = 1), government health system staff (*n* = 5), government front line field workers (*n* = 22), and community members (*n* = 2). The assessment was based on: workforce, service delivery, financing, governance, information system, medical supplies, and the broad socio-political context.

**Results:**

The government of Bangladesh has developed inpatient and outpatient guidelines for the management of SAM. There are cadres of community health workers of government and non-government actors who can be adequately trained to conduct CMAM. Inpatient management of SAM is available in 288 facilities across the country. However, only 2.7% doctors and 3.3% auxiliary staff are trained on facility based management of SAM. In functional facilities, uninterrupted supply of medicines and therapeutic diet are not available. There is resistance and disagreement among nutrition stakeholders regarding import or local production of ready-to-use therapeutic food (RUTF). Nutrition coordination is fragile and there is no functional supra-ministerial coordination platform for multi-sectoral and multi-stakeholder nutrition.

**Conclusion:**

There is an enabling environment for CMAM intervention in Bangladesh although health system strengthening is needed considering the barriers that have been identified. Training of facility based health staff, government community workers, and ensuring uninterrupted supply of medicines and logistics to the functional facilities should be the immediate priorities. Availability of ready-to-use therapeutic food (RUTF) is a critical component of CMAM and government should promote in-country production of RUTF for effective integration of CMAM into the health system in Bangladesh.

**Electronic supplementary material:**

The online version of this article (10.1186/s12913-018-3087-9) contains supplementary material, which is available to authorized users.

## Background

Severe acute malnutrition (SAM) is the most serious form of childhood acute malnutrition and is associated with very high rates of morbidity and mortality. Although rarely documented as a direct cause, more than 50% of all childhood deaths are attributable to undernutrition, with relative risks of mortality being 8.4 for severe acute malnutrition, and 4.6 for moderate acute malnutrition (MAM) [1]. Globally approximately 19 million children under 5 years are affected by SAM [2, 3]. The World Health Organization (WHO) recommends inpatient treatment regimes of intensive medical and nutritional protocols for the management of SAM. However, limited inpatient capacity, high cost, low number of trained staff available in the hospital result in low coverage, low recovery, high mortality and high defaulting at the inpatient therapeutic programs [4, 5]. This has led to the innovation of community based management of acute malnutrition (CMAM). CMAM is an integrated public-health approach to address acute malnutrition emphasizing treating uncomplicated SAM patients solely as outpatients while keeping only the complicated SAM cases as inpatients [6]. Effectiveness of CMAM in emergencies is well documented in Ethiopia, Malawi, South Sudan, North Sudan, and Niger [7–9]. In the non-emergency contexts, for sustainability and long-term improvements in the management of SAM require that CMAM be implemented through the existing ministry of health infrastructures as a part of a standard primary health care package [10, 11].

Bangladesh has 8 administrative divisions divided into 64 districts. These districts are further divided into sub-districts or upazilas, unions and wards. The ministry of health and family welfare through the directorate general of health services (hereafter referred as DG health) and directorate general of family planning (hereafter referred as DG family planning) operates a dual system of health and family planning service delivery through medical college hospitals, specialized hospitals, district hospitals, upazila health complexes (with 51 beds), maternal and child welfare centres, union health and family welfare centres, union sub-centres and community clinics [12]. Community clinics were established to provide integrated health and family planning services from a single primary health care centre at the village level. DG health and DG family planning have their own cadre of field level workers i.e. health assistants (HA) and family welfare assistants (FWA), respectively to provide domiciliary services. Community clinics are run by community health care providers (CHCP) assisted by HAs and FWAs.

CMAM in Bangladesh has been implemented at a limited scale by several non-government agencies as an emergency response to natural disasters [13, 14]. Considering the case load of acute malnutrition, the government has prioritized integration of CMAM into the health system in the 4th health population and nutrition sector program (January 2017–June 2022) [15]. Therefore, it is important to assess the readiness of the health system functionaries with regard to identification of malnourished children in the community, distribution of therapeutic foods, and follow up of the treated cases. Choudhury et al. has reported the feasibilities and constraints of CMAM implementation in Bangladesh [16]. However, the article was essentially a desk review and did not systematically analyze the multiple dimensions of health system in the context of CMAM implementation [17]. Likewise, Kouam et al. has reviewed the health system integration of CMAM in two sub-districts in Southern Bangladesh in a setting where an international non-government organization (NGO) was implementing CMAM [14]. The present study attempted to assess the health system readiness to implement CMAM considering the Northern and Southern regions of Bangladesh, and based on the WHO health system framework [17]. It also includes an analytical discussion on enabling factors and constraints of the current health system in Bangladesh.

## Methods

### The conceptual framework

We based our assessment on a framework that encourages systems thinking incorporating the WHO health system building blocks [18], and also encompassing the broad socio-political context that affects integration of an intervention [19]. The elements of the framework were: i. broad context of policies and programs ii. features of CMAM intervention, iii. Health system characteristics, and iv. qualities of the adoption system [19].

### Data collection

**Fig. 1:**
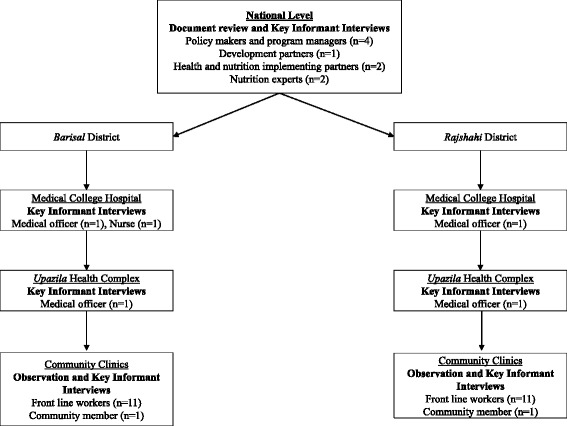
Data collection procedure

Document review, key informant interviews and direct observations were employed as depicted in Fig. 1. The study took place during January to May 2014 at the national level and in one Southern (Barisal) and in one Northern (Rajshahi) district in Bangladesh. In response to floods or cyclones, several international NGOs implemented CMAM in Barisal. In Rajshahi there were modest NGO activities addressing acute malnutrition, although the district is well known to suffer from seasonal food insecurity. In each district data were collected from one upazila health complex, three community clinics, and Rajshahi and Barisal medical college hospitals. One well-performing (according to the government’s report), 1 hard-to-reach, and 2 peri-urban community clinics were randomly selected (Fig. 1).

#### Document review

We reviewed documents pertaining to the national health system, child health and nutrition services and the ongoing programs at the national, district and sub-district levels. The documents were collected from official sources including the ministry of health and family welfare, institute of public health nutrition and community based health care directorate, NGO offices, health workers and local health administration offices. The reviewed documents included national nutrition policy 2015, operational plans of national nutrition services and community based health care, the sixth five year plan of the government, health, nutrition and population sector development program document, the government guideline on CMAM, government reports on nutrition service delivery, and CMAM implementation reports of NGOs. We also did literature search in PubMed on the management of acute malnutrition focusing on Bangladesh.

#### Key informant interviews

A total of 38 key informant interviews were conducted among the key individuals involved in child health and nutrition program design and implementation in the country. The selection of participants was purposive depending on their involvement in child health and nutrition policy and program implementation in Bangladesh. The respondents were government policy makers and program managers (*n* = 4), nutrition experts (*n* = 2), health and nutrition implementing partners (*n* = 2), development partner (*n* = 1), government health system staff (*n* = 5), government front line field workers (*n* = 22), and community members (*n* = 2). Topics covered during the interviews were derived from the study themes that were based on the WHO health system framework [18]. Separate interview guidelines were prepared for different types of respondents (Additional file 1). The interview sessions were conducted by investigators involved in the study and were conducted in *Bangla* and English as appropriate. Informed consents were obtained before the interview sessions, and all interviews were audio-taped.

#### Direct observation

The related activities of selected community clinics, upazila health complexes, and medical college hospitals were observed to collect information on nutrition service delivery, available equipment and supply chain. Such observational reviews in each facility lasted for at least 4 h using a set of check lists for consecutive 9 days in each study area.

### Data analysis

We followed thematic analysis approach to organize and interpret responses. English transcripts were developed based on all audio-recorded interviews. The transcripts were analyzed thematically following the conceptual framework [19]. The pre-defined themes were divided into sub-themes and transcripts were reviewed to develop code list for the topics related to the conceptual framework and the WHO health system building blocks. Triangulation of information gathered from different sources was conducted to examine validity of the information. Health system situation suitable for CMAM integration was considered based on provisions of grass roots level service centres, human resources, logistics, and an enabling adopting system.

### Ethical clearance

Approval for the study was obtained from the Institutional Review Board of icddr,b. Informed written consents were obtained from the participants. Confidentiality of the information provided was maintained at all stages of data analysis.

## Results

### The broad context

#### Nutrition programs, policies and guidelines

Bangladesh implemented vertical nutrition programs, i.e. Bangladesh Integrated Nutrition Project and National Nutrition Program during 1996 to 2011 where reducing national rates of acute malnutrition was one of the priorities. Considering the importance of an integrated approach, nutrition services were integrated within the existing ministry of health and family welfare service delivery systems as national nutrition services (NNS) since July 2011. The institute of public health nutrition under the ministry of health and family welfare was made responsible to implement NNS including inter alia CMAM and ensuring coordination between different ministries for nutrition interventions.

The government has endorsed the national nutrition policy 2015 and the second national plan of action for nutrition (2016–2025). The policy puts much emphasis on treating severe and moderate acute malnutrition both at health centre and in the community. Institute of public health nutrition developed the national guideline for facility based management of SAM in 2008 [20]. A national guideline for the community-based approach to manage acute malnutrition was published in 2011 to complement the national inpatient SAM management guideline [21].

### Features of CMAM intervention

**Fig. 2:**
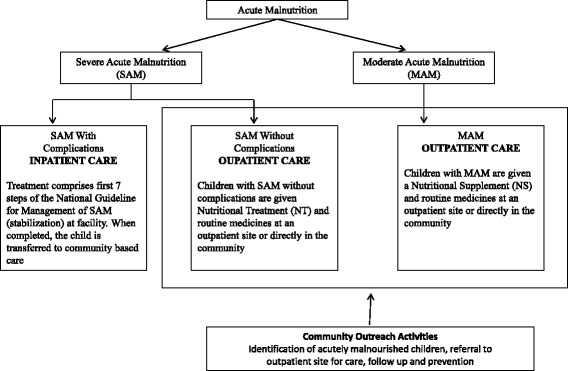
Features of CMAM intervention in Bangladesh

The national guideline for CMAM in Bangladesh adopts the global CMAM approach to the local context. Figure 2 depicts the features of CMAM intervention as outlined in the national guideline [21]. The guideline emphasizes on identifying children with acute malnutrition at the community or household levels using MUAC tapes by the front line community health workers (CHW), i.e. HA (DG health cadre), FWA (DG family planning cadre), CHCPs, and NGO community health or nutrition workers. Growth monitoring and promotion, routine vaccination, expanded program on immunization campaign, community clinic visits or home visits by CHWs are platforms to be used for identification of malnourished children. The uncomplicated cases can be managed by CHW by using nutritional and/or medical treatment. The serious and complicated cases are to be referred to the outpatient sites where the trained CHW will assess whether the child requires inpatient care and accordingly will refer to the upazila health complexes [21]. Inpatient care for stabilization is to be provided to SAM children with complications following the national guideline [20]. The national CMAM guideline places much emphasis on prevention of childhood malnutrition by behavior change communication, and follow up of children who received treatment for acute malnutrition at the community level.

### Health system characteristics for implementing CMAM

The health system characteristics of workforce, service delivery, medical products, financing, information system, and governance were assessed in relation to CMAM implementation in Bangladesh [18].

#### Workforce

The national CMAM guideline envisages the government front line CHWs to conduct community level screening for malnutrition in children. Review of documents revealed that existing CHWs, i.e. HAs and FWAs were tasked to work fulltime 6 days/week to complete their domiciliary duties. However, after establishment of community clinics, they are assigned to work 3 days/week at the clinics without any modification in their existing domiciliary responsibilities and targets. When interviewed, HAs and FWAs expressed their dismay regarding time constraints for home visits under current work schedule. Similar finding was obtained in key informant interviews with policy makers.

Document review revealed that Bangladesh has 3.92 CHW per 10,000 population [22]. Total number of ministry of health and family welfare community-level health workers relevant for CMAM implementation is around 46,000, although there are many vacant positions [22]. Table 1 depicts the numbers of actual and vacant posts of all grassroots level cadres relevant to CMAM in 2014 [22]. Besides, adequately trained cadre of CHW is very important to implement CMAM. The CHWs interviewed in the study also reported that they did not receive training on identification or management of acute malnutrition. Review of documents revealed that the nutrition training modules prepared for the CC staff did not contain any section on CMAM or management of acute malnutrition. NNS provides training on facility based management of SAM as well as CMAM. Until 2014, NNS has trained government health cadres in 45 upazilas (out of a total of 492 upazilas in Bangladesh) on SAM management. Table 2 depicts proportion of physicians who received training on public health nutrition in past 3 years as of 2011 [23]. The findings revealed that, on average, only 2.7% physicians and 3.3% auxiliary staff were trained on management of severe acute malnutrition.

**Table 1:** Number of sanctioned and vacant posts of government community health workers in 2014

Cadre title	Numbers of sanctioned position	Number of staff appointed	Number of vacant positions
FWA	23,500	21,083	2417
HA	20,877	17,532	3345
CHCP	13,861	13,822	39

**Table 2:** Proportion of doctors and auxiliary staff receiving training on child health and nutrition as of 2011 [23]

	Doctors (%)				Auxiliary staff (%)			
Health facilities	IMCI^*^/CIMCI^**^	SAM management	General nutrition for children	Causes/prevention of malnutrition	IMCI/CIMCI	SAM management	General nutrition for children	Causes/prevention of malnutrition
DH^a^	6.8	2.2	4.4	4.4	2.6	0	7.9	10.5
UHC^b^	19.1	2.6	6.8	6.6	21.5	2.5	6.3	10.1
MCWC^c^	7.6	4.6	5.3	5.3	3.8	0	1.9	3.8
UnHFWC^d^	0	0	16.6	0	13.6	2	7.3	6.6
USC^e^	16.3	5.4	5.4	10.8	20.8	0	2.9	5.8
CC^f^	–	–	–	–	11.6	6	8.4	8.8
All	14.7	2.7	6.7	6.0	17.4	3.3	6.9	9.5

*IMCI integrated management of childhood illness, **CIMCI community based integrated management of childhood illness, ^a^*DH* district hospital (including medical college hospitals), ^b^*UHC* upazila health complex, ^c^*MCWC* maternal and child welfare centre, ^d^*UnHFWC* union health and family welfare centre, ^e^*USC* union sub-centre, ^f^*CC* community clinic

#### Service delivery

The NNS officials informed that instructions had been given to the upazila health complexes where SAM corners are available to engage the grass-root level staff in screening malnourished children by using MUAC tape. During observation, we did not find any government worker in those areas who were actively engaged in screening for identifying malnourished children as they did not receive training, or MUAC tapes to identify children with acute malnutrition. We observed that nutrition counselling of mothers was a regular activity of CHCPs, HAs, and FWAs. Counselling was primarily limited to promotion of exclusive breastfeeding, care during pregnancy, healthy dietary habits and hygiene practices. One of the community clinic staff member reported that she could only detect malnutrition through observation but was unable to quantify its severity.

*“I know about malnutrition. Many children are born with malnutrition. We don’t measure the weight, but we can perceive the child’s nutritional status by taking the baby in the lap”* indicated by a FWA.

In Barisal district we found an international NGO active in implementing CMAM through community clinics. A CHCP trained by them was found identifying children with acute malnutrition using MUAC tape, administering antibiotics following the protocol and referring complicated cases to the respective upazila health complex where the SAM corner was run and supported by that NGO. Active case finding by ‘door-to-door’ screening was being conducted by trained community health workers employed by the NGO. Other community clinics that we visited were not supported by the NGO, and did not conduct any such activity related to CMAM.

Key informant interviews revealed that NNS has established SAM corners in 288 facilities including medical college hospitals, district level hospitals and upazila health complexes. Rajshahi and Barisal medical college hospitals had specialized SAM corners for inpatient treatment of SAM with 5 and 8 beds, respectively. In both hospitals, SAM children were primarily identified at the outpatient facilities. However, in Barisal, an international NGO implementing CMAM referred SAM children to the medical college hospital.

#### Health information system

Nutrition related indicators have been incorporated within the existing Management Information System (MIS) of DG health and DG family planning. The nutrition register books maintained at the community clinics included information on height, weight, MUAC and severe acute malnutrition. Observation and key informant interviews revealed that all clinics were equipped with laptops and internet connection, and the CHCPs reported the service delivery statistics online. The statistician at upazila health complex feeds the field level information to national health data repository. However, all the government CHWs were observed to have inadequate training on measuring nutritional status of children and to be able to fill in the register books correctly. Interview with CHW revealed that the CHWs interviewed did not receive satisfactory training on assessing nutritional status, and filling in the register books for nutrition. During interview, program manager from NNS also expressed his concern regarding the quality of nutrition data that came from field due to the fact that the training of CHWs was inadequate. It was also observed that community clinic care seekers, particularly malnourished or sick children, did not have any registration number or identification number to track the child, monitor his improvements or compliance to follow-up visits.

#### Medical logistics

The national CMAM guideline emphasizes on treating children with uncomplicated acute malnutrition by using ‘Nutrition Treatment’ (which is a nutrient dense food supplement); specification of which matches with the ready-to-use-therapeutic foods (RUTF) internationally available. The guideline also mentions that the children should also receive vitamin A, broad spectrum antimicrobial amoxicillin, albendazol and measles vaccine [21]. The ‘stabilization’ phase of CMAM (inpatient care) is a 7-step management protocol and requires glucose/sucrose (either orally or through infusion if hypoglycemic), Rehydration Solution for Malnutrition (ReSoMal), broad spectrum antibiotics, and the therapeutic milk-based diets, i.e. F-75 and F-100 [20].

RUTF was unavailable in the visited facilities in both study areas. Data from key informant interviews revealed that international NGOs working on CMAM used imported RUTF, i.e. Plumpy’nut in their program areas. In visited community clinics we found stocks of certain antibiotics which were relevant to treat complicated SAM children.

In the medical college hospitals we found stocks of F-75 and F-100. In one of the hospitals, key informant interview revealed that there have been many instances when they were out of F-75 and F-100 in stock as they get the supplies at every three to four months intervals. In case of unavailability, the hospital made the therapeutic diet locally by using rice, milk, sugar and oil. ReSoMal was available in one of the medical college hospitals. However, vitamin and mineral mix required for treatment of SAM children when F-75 and F-100 are out of stock was not available. Key informant interview revealed that that in cases of non-availability of hospital supplies, particularly medicines, the parents are usually advised to buy the medicines from outside through out-of-pocket payment.

#### Financing

The total budget of current health, population and nutrition sector program (January 2017 to June 2022) is USD $14.71 billion [15]. For NNS, allocated budget is USD $92.88 million (6.58% of total health, population and nutrition sector program budget) of which around USD $3.08 million has been allocated for the management of children with moderate and severe acute malnutrition (3.3% of total NNS budget). This budget includes costs of relevant guideline/training module development, staff training and procurement of supplies [15].

#### Governance

‘Governance’ can be interpreted in different ways. We used it to refer to nutrition coordination mechanism that exists in the country. Bangladesh national nutrition council was first established in 1975, and later on revitalized in 2016. The council is responsible to provide guidelines on nutrition, and coordination of nutrition activities across ministries. The council is yet to be fully functional in July 2017. Nutrition cluster in Bangladesh was activated in 2007 with representations from over 15 member organizations including UN agencies, international and local NGOs, national institutes and research/academic institutions. UNICEF and institute of public health nutrition under ministry of health co-chair nutrition cluster. Nutrition cluster is the focal point for scale up of management of childhood SAM. Besides, the nutrition working group is a forum of stakeholders discussing and sharing emerging issues related to nutrition. A CMAM working group, though initiated under nutrition working group during 2010, was not functional during the time of assessment.

### Adopting system

The study explored the perceptions of key health actors with varied interests, and power distribution to understand the facilitating factors or constraints of CMAM integration (Fig. 1). The findings are summarized below:

#### Perceptions of policy makers and health managers

##### Facilitating factors

The current health system structure is favorable to implement CMAM. At the village level, there are 13,136 functional community clinics with a supply of 29 types of medications including certain antibiotics. Each community clinic serves around 6000–10,000 population. In certain upazila health complexes, integrated management of childhood illness (IMCI)-nutrition corners have been established, at which complicated SAM cases can be treated. Doctors and nurses are trained on facility-based management of SAM in primary, secondary and tertiary level health facilities. For community service, there are existing cadres of domiciliary workers (i.e. HA and FWA) who can be utilized to implement CMAM. The nutrition information and planning unit at NNS is working towards incorporating standard nutrition indicators into the health MIS. Additionally, there is auxiliary power of NGOs who are working in the field of nutrition, particularly CMAM, in areas with high burden of childhood malnutrition. The government recognizes the need for greater involvement of the private sectors for enhancing effective health service delivery; public private partnership in service delivery is particularly encouraged.

*The government alone cannot eradicate our health and nutrition problems, we do need the auxiliary power of the NGOs and all of our achievements in our health sector have been derived from GO-NGO partnership and we have to keep on working together ……*mentioned by a policy maker in DG health.

##### Constraints

Only facility-based management of SAM in children is present in Bangladesh. However, well-functioning referral system is not available everywhere. This was also found during direct observation. Although training on SAM management is ongoing, there is still shortage of trained human resources (i.e. doctorsand nurses) for facility-based management of SAM. Although IMCI- nutrition corners are functional in some facilities, only complicated children get admitted, and preventive nutrition counseling is not feasible. This was also found during direct observation of the IMCI-nutrition corners. There is inherent lack of coordination between health and family planning wings of the government health system. Besides, there are no dedicated human resources for NNS to implement nutrition interventions. Moreover, frequent transfer of doctors with training on SAM management from present place of assignment is a concern for optimal in-patient service delivery. For children with moderate acute malnutrition, counseling is the only intervention that is available. The most fundamental constraint of implementation of CMAM in Bangladesh is the unavailability of RUTF. There is strong resistance, not supported by any evidence, from few stakeholders on importing RUTF or producing it locally. This resistance is impeding work on CMAM in the country, both in the public and NGO sectors.

### Perceptions of service providers

#### Facilitating factors

The CHCPs who are the new cadre at community clinics work 6 days/week in the clinic. They can be trained to detect children with malnutrition using MUAC tape and manage uncomplicated SAM cases by providing RUTF, counseling, and antibiotics as necessary. HAs and FWAs can identify cases of acute malnutrition during their domiciliary visits. Medical officer responsible for public health and nutrition at upazila health complexes can provide overall supervision and guidance for CMAM implementation.

#### Constraints

Training of CHCPs, HAs, and FWAs was not adequate to implement CMAM. HAs and FWAs were already burdened with their existing responsibilities; now nutrition service at community clinics was perceived as ‘additional work’. There is dissatisfaction among the HAs and FWAs regarding higher salary scale of CHCPs.

*There is a difference in terms of wage scale between us and the CHCPs. Being new in service they are getting a monthly salary which is more than us but we are in service for a much longer period than them. So, this different salary structure is an unacceptable problem and causing dissatisfaction among us in our workplace and despite this disparity we are continuing to work* ……..mentioned by a FWA.

## Discussion

**Fig. 3:**
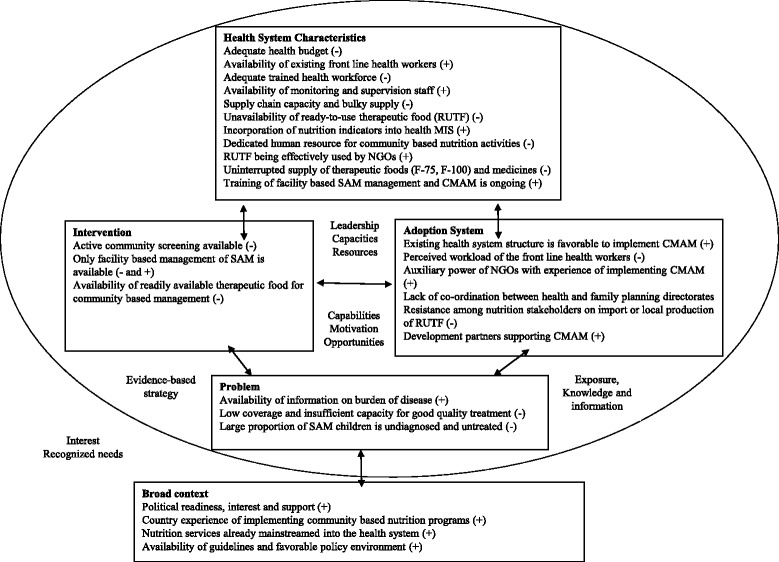
Factors influencing integration of CMAM into health system in Bangladesh

The study provided insights into the factors that would influence the implementation of community based management of children with acute malnutrition through the national health system in Bangladesh. Figure 3 presents a synthesis of the key findings. Improving child nutrition is a government priority, and the national nutrition program (i.e. the national nutrition services) and nutrition policy foster potential integration of CMAM into the health system [24]. The UN joint statement recommends that it is fundamental to have a national protocol prior to the training of health workers on CMAM interventions delivered through the health system [10]. Bangladesh has made a remarkable progress in this regard; the government has already developed national guideline for CMAM. Treatment of SAM children is being operational in 288 health facilities across the country. In countries where CMAM integration has been successful like Ethiopia, Sierra Leone, Malawi, Nepal, and Ghana, the activities were closely linked to antenatal care, infant and young child feeding (IYCF) practices, immunization, IMCI, and growth monitoring interventions [25]. In Mozambique, CMAM is integrated into the reproductive and maternal child health services, HIV/AIDS and tuberculosis services, health promotion, and community involvement activities. In Kenya, most outpatient treatment services for SAM management are located in the Maternal and Child Health clinics [25]. Similar approach can be adopted in Bangladesh for successful implementation of CMAM through the health system. At present, inpatient management of acute malnutrition is integrated with IMCI services in Bangladesh. Table 3 presents a comparison of Bangladesh context with experiences of selected countries successfully implemented CMAM nationally.

**Table 3:** Country experiences of successful CMAM programs nationally [31, 37]

Indicators	Ethiopia	Kenya	Malawi	Nepal	Pakistan	Bangladesh
Health system characteristics						
Adequate health budget	> 75% donor support	> 80% donor support	> 90% donor support	> 50% donor support	Human resources and infrastructure supported by government	Overall budgetary allocation for nutrition is poor
Availability of community based front line workers	Good	Good	Weak	Weak	Good	Inadequate; not utilized for CMAM
Nutrition indicators in health MIS	No	Yes	Yes	No	Yes	Yes
Supply of RUTF, F-75, F-100	Weak national supply chain	F-75, F-100 supply good. Interrupted supply of RUTF	Good. Local production of RUTF	Good	Interrupted supply of RUTF at times	Sensitivity around RUTF, interrupted supply of F-75, F-100
CMAM services integrated with other health services	^*^IMNCI, ^**^ICCM, immunization	^a^IMCI, ^b^IYCF	HIV/AIDS	IMCI, IYCF	Unclear	IMCI
Intervention components						
Active screening by government staff	Good	Good	Good	Good	Yes	Weak
Inpatient care by WHO protocol	Yes	Yes	Yes	Yes	Yes	Yes
Community mobilization	Good	Good	Good	Good	Good	Weak
Management of MAM	Weak	> 80% recovery rate	89% recovery rate	Weak	> 90% recovery rate	No services until now
Program outcomes (outpatient)						
Recovery (SPHERE standard, > 75%)	83.0%	80.7%	91%	86.1%	91.5%	No national program
Default (SPHERE standard, < 15%)	5.0%	12.9%	6.0%	9.0%	7.5%	No national program
Mortality (SPHERE standard, < 10%)	0.6%	1.5%	1.0%	0.7%	0.2%	No national program
Broad Context						
National policy support	Yes	Yes	Yes	Yes	Yes	Yes
National guideline on CMAM	Yes	Yes	Yes	Yes	Yes	Yes
Donor supporting CMAM	Yes	Yes	Yes	Yes	Yes	Yes
NGOs implementing CMAM	Yes	Yes	Yes	Yes	Yes	Yes

^*^IMNCI Integrated management of neonatal &childhood illnesses, **ICCM integrated community case management, ^a^*IMCI* Integrated management of childhood illness, ^b^*IYCF* Infant and young child feeding

The existing health system pyramid is very favorable for CMAM incorporation; the system provides infrastructure up to the ward or village levels. Bangladesh is one of the very few countries that has successfully scaled up and sustained its community level workforce [26]. There are cadres of government CHWs, i.e. HAs and FWAs providing domiciliary services. HAs and FWAs visit the households as part of their routine job, which is an opportunity to include CMAM outreach services in their activities. Besides, there is an auxiliary workforce of NGO yet untapped for implementation of CMAM [26]. In this regard, the CHWs would have to be trained and provided with MUAC tapes, referral slips and sensitization materials such as flyers or pamphlets containing related behavior change communication messages on prevention of childhood acute malnutrition. CMAM outpatient services can be provided from the community clinics, upazila health complexes, union sub-centres, union health and family welfare centres, and NGO health centres. To achieve this objective, alongside training of the health staff, these facilities would need to be made more functional by ensuring supply of anthropometric and medical materials, improved storage facilities, and uninterrupted supply of medicines and therapeutic foods. However, utilization of the front line health workers or operationalization of DG family planning facilities for CMAM services will be challenging due to the intrinsic tension between ‘health’ and ‘family planning’ directorates of the ministry of health and family welfare [27]. Additionally, the workload of the DG family planning front line workers is also a limiting factor for utilizing them for CMAM.

Shortage of trained staff is a critical challenge that the health system will be facing while implementing CMAM through the existing mechanism. The study identified that only 14.2% and 2.7% doctors are trained on IMCI and SAM management, respectively. The corresponding proportion for auxiliary staff is 17.4% and 3.3%, respectively. However, irrespective of trained health personnel on childhood malnutrition management, Bangladesh has a critical shortfall of qualified healthcare providers. The health care system only has around 5 physicians and 2 nurses for every 10,000 population as opposed to the 23 health professionals for every 10,000 people, as considered adequate by WHO [26, 28]. However, shortage of health workers is a common problem in most countries in Southeast Asia [29]. Similar assessment conducted in Bangladesh has reported that health workers prefer to work in cities and for NGOs because of better working conditions and attractive pay packages [14]. Analogous to this, the present study identified dissatisfaction among HAs and FWAs regarding higher salary scale of CHCPs. The study also found that frequent transfer of human resources who were trained on SAM management from the present working place is one of the important constraints of CMAM implementation. For successful implementation of CMAM, it is critical to endorse an appropriate strategy for proper distribution and retention of trained health personnel in the functional facilities.

A study carried out in Burkina Faso showed that 77% of the children suffering from acute malnutrition were successfully managed in the community [30], and in Nepal and Pakistan community based management was successful in curing 80% of the cases [31]. The ministry of health and family welfare of Bangladesh should now invest more in community based management of acute malnutrition to reduce the inpatient load of SAM cases and improve early case detection, and thereby considerable reduction in death rates. The NGOs in Bangladesh have consistently trained and deployed community health workers effectively throughout the country [32]. The current numbers of grass-root level NGO health workers is about three times higher than that of government health system [26]. Recent studies conducted in southern Bangladesh showed that community workers were able to provide quality care in managing cases of SAM without complications; provided that they received good training and regular supervision [33]. The intervention achieved high coverage, low defaulting, high recovery and low mortality, and was cost-effective [13, 34]. To counterbalance the shortage of government health system staff, involving NGO community workers in CMAM implementation in Bangladesh would be an effective strategy. This would eventually reduce the workload of the existing CHWs.

In successful countries the two main factors that facilitated successful integration of CMAM were (1) the political commitment and leadership of the government for the implementation of primary health care package, in which they have included CMAM activities, and (2) good coordination between the government, NGOs and donors for strategizing integration within the health system and scaling up of CMAM services [25]. In Bangladesh there are mechanisms to oversee implementation of nutrition services operated by government policy bodies. These mechanisms complement other nutrition coordination forums like UN Ending Child Hunger and Undernutrition, and Scaling Up Nutrition movement. However, coordination is fragile and overlapping of activities and confusion still exist in the area of nutrition service delivery. Although the ‘health’ and ‘food’ sectors have frameworks for nutrition related activities, there is no functional supra-ministerial coordination platform for multi-sectoral and multi-stakeholder nutrition services in Bangladesh as exists in other countries, i.e. Senegal, Peru, Brazil and Nepal who have success in scaling up nutrition services [35]. Furthermore, in Bangladesh, there is strong resistance and fragmentation among nutrition stakeholders regarding local production or import of RUTF. Although the national guidelines recommend use of locally produced therapeutic foods, the ministry of health and family welfare is yet to approve the use of commercially available RUTF for community based management of SAM. The disagreement is primarily due to i. stance against processed food, ii. non-local foods, iii. breast milk substitution, iii. economic implications and iv. commercialization of efforts addressing childhood malnutrition [36].

The situation analysis in only two districts, and the fact that respondents were selected purposively are two potential limitations of the study. The findings are, therefore, not representative of the whole country, and this may hamper the external validity of the study. Nevertheless, the analyses framework that has been used in the study provided useful observations on health system preparedness, highlighting the areas that need strengthening prior to, and during implementation of CMAM.

## Conclusion

The assessment of present health system revealed that despite certain shortcomings, there is an enabling environment for implementing CMAM in Bangladesh. Training of facility based health staff, government and non-government community workers for outpatient management of severe acute malnutrition cases, and ensuring uninterrupted supply of medicines and logistics to the functional facilities should be the immediate priorities. Furthermore in-country production of RUTF using locally available ingredients is critical for ensuring community case management and effective integration of CMAM into the health system in Bangladesh.

## Additional file

Additional file 1: Key Informant Interview Guidelines. (DOCX 29 kb)

## Abbreviations

CHCP: Community health care provider
CHW: Community health workers
CMAM: Community based management of acute malnutrition
FWA: Family welfare assistant
HA: Health assistant
IMCI: Integrated management of childhood illness
MAM: Moderate acute malnutrition
MUAC: Mid upper arm circumference
RUTF: Ready-to-use therapeutic food
SAM: Severe acute malnutrition
