# Supplementary material for: Challenges and opportunities of integration of community based Management of Acute Malnutrition into the government health system in Bangladesh: a qualitative study
Source: BMC Health Serv Res. 2018 Apr 10;18:256. doi: 10.1186/s12913-018-3087-9 (PMC5892001; doi:10.1186/s12913-018-3087-9)
Supplement: Supplementary file 1 — Key Informant Interview Guidelines. (DOCX 29 kb) [file 12913_2018_3087_MOESM1_ESM.docx]

**Additional file 1: Key Informant Interview Guidelines**

**A. Government policy makers and program managers**

**1. Service Delivery**

- What services are you currently providing for severe and moderate acute malnutrition of children at facility level and community level? How are you providing these services?
- Can you please give us an idea of NNS coverage and utilization of services for SAM, MAM last year?
- Please briefly tell us about quality control measures you intend to follow for CMAM delivery?
- Please tell us about NNS’s partnership/collaboration with NGOs working in respect of CMAM?
- Please tell us about your plan to scale-up Community-based Management of Acute Malnutrition (CMAM) service. What is the plan for providing Nutrition Treatment and Nutrition Supplements for SAM and MAM children in the community?
- Please tell us whether the current community outreach activities of community clinics for screening pregnant and lactating mothers is capable of effective screening children with SAM and MAM
- Please tell us about NNS’s plan for counseling of caregivers of SAM and MAM children at community level
- Please tell us about SAM corners at upazila health complexes.

**2. Work Force**

- Did you do any workforce assessment for implementing CMAM or NNS as a whole? (If yes) Please tell us about it
- Please tell us about the adequacy of the current workforce dedicated for CMAM and inpatient treatment for SAM
- Please tell us about different trainings you are currently providing for capacity building of government staff members relevant to CMAM and SAM.
- What other trainings have you planned to provide in future relevant to CMAM and inpatient SAM management.
- Please tell us how you are ensuring accountability of the staff dedicated to SAM treatment at UHCs and CMAM at community level.
- Please tell us about the workload distribution of CHCP, HA, FWA and Community Group members in respect of CMAM.

**3. Information**

- May we know how do you create mass awareness on SAM and MAM?
- How do you compile and analyze NNS service data reported separately by health and family planning staff at CC level?
- Please tell us about the problems you are facing regarding health MIS.
- How much of MIS data on NNS are being used for planning, monitoring and evaluation?

**4. Medical Products**

- We have identified F 75, F 100, RESOMAL, therapeutic foods and antibiotics for treating SAM at upazila health complexes, and SAM and MAM at community level. Please tell us About NNS’s perception and plan to integrate this products into CMAM and their cultural sensitivity.
- Please tell us how do you procure and distribute them.
- Please tell us about the storage facilities available for medical products at community clinics, upazila health complexes, medical college hospitals and district hospitals.

**5. Financing**

- Would you please brief us about sources of funding for NNS particularly CMAM?
- Do you think it is adequate? If no, please explain.
- Can you please tell us the estimated cost per child for inpatient SAM treatment and for CMAM services? How can you minimize the cost?
- We heard that fund release for CMAM has been delayed. Please tell us why and what could be done in future to prevent it
- Please tell us about the role of World Bank in terms of fund disbursement for NNS and CMAM

**6. Leadership/Governance**

- What is your observation about mainstreaming of NNS with the existing service delivery system? Please elaborate the strengths, weaknesses, threats and opportunities of this mainstreaming approach
- Would you please brief us about the intersectoral, interministerial and development partners coordination mechanism for CMAM implementation?
- Please tell us about the relevance and roles of nutrition implementation committee and civil society groups for implementation of CMAM.

**B. Development Partners**

- In context of implementing CMAM by the government, please tell us about the existing roles of development partners.
- What are the barriers to implement CMAM in Bangladesh?
- Do you think that current workforce in CCs are adequate for delivering CMAM services effectively? Please explain.
- How therapeutic foods like F75, F100, RASOMAL and RUTF could be provided within the package of CMAM?
- How development partners can help to mainstream RUTF for CMAM?
- What should be the role of development partners in in persuading government to allocate funds for CMAM?
- How the development partners coordinate and collaborate with the government and NGOs for implementing CMAM?
- What is your institution’s plan in the future for CMAM?
- What is your observation about mainstreaming CMAM with the existing service delivery system? Please elaborate the strengths, weaknesses, threats and opportunities of this mainstreaming approach.

**C. Health and Nutrition Implementing Partners**

**Service Delivery**

- In context of implementing CMAM by the government, please tell us about the existing roles of NGOs.
- Please tell us what further things NGOs are capable of doing to aid government to implement CMAM.
- What are the barriers to implement CMAM in Bangladesh?
- Please tell us about the capability of existing health care delivery system of Bangladesh to integrate and scaling up of CMAM program?

**2. Work Force**

- Do you think that current workforce in community clinics are adequate for delivering CMAM services effectively? Please explain.
- What type of training do you think would be helpful for the service providers to deliver effective CMAM services?

**3. Information**

- What steps should be taken to create mass awareness on SAM & MAM?
- How the referral system could be improved?
- Do you think the monitoring & supervision in community clinics are enough to ensure accountability? If not, please explain what could be done to strengthen the monitoring system.

**4. Medical Products**

- How therapeutic foods like F75, F100, RASOMAL and RUTF could be provided within the package of CMAM?
- How NGOs can help to mainstream RUTF for CMAM?
- Do you think any other drugs needed to be added in the free drug list provided by community clinics for CMAM? Please explain.

**5. Financing**

- Can you please tell us the estimated cost per child for inpatient SAM treatment and for CMAM services? How can we minimize the cost?
- What should be the role of NGOs in persuading government and development partners to release funds for CMAM?

**6. Leadership/Governance**

- How the NGOs coordinate and collaborate with the government and other development partners for implementing CMAM?
- What is your institution’s plan in the future for CMAM?
- What is your observation about mainstreaming CMAM with the existing service delivery system? Please elaborate the strengths, weaknesses, threats and opportunities of this mainstreaming approach.

**D. Nutrition Experts**

- In context of implementing CMAM by the government, please tell us about the existing roles of NGOs.
- What are the barriers to implement CMAM in Bangladesh?
- How the inpatient service delivery for SAM children at upazila health complexes, zila hospitals and medical college hospitals could be improved?
- Please tell us about the capability of existing health care delivery system of Bangladesh to integrate and scaling up of CMAM program?
- Do you think that current workforce in CCs are adequate for delivering CMAM services effectively? Please explain.
- How NGOs can help to mainstream RUTF for CMAM?
- How the NGOs coordinate and collaborate with the government and other development partners for implementing CMAM?
- What is your observation about mainstreaming CMAM with the existing service delivery system? Please elaborate the strengths, weaknesses, threats and opportunities of this mainstreaming approach?

**E. Government health system staff**

**1. Service Delivery**

- What services are you currently providing through this facility?
- What services do you provide for maternal and child nutrition?
- What services are available for children with severe acute malnutrition and moderate acute malnutrition?
- What medications/food do you provide for malnourished children?
- What medications/food do you provide for malnourished children?
- Please tell us about follow-ups and referral from the community

**2. Work Force**

- How many people work here for the management of malnourished children? What are your job responsibilities?
- Please tell us what special trainings (if any) you have received about managing malnourished children.
- Does this facility have adequate human resource for the management SAM children?
- Please tell us about role of existing NGOs in aiding you with your activities.

**3. Information**

- Have you received any training on how to collect and record data?
- Please tell us how you report service related data?
- Please tell us about problems you encounter regarding data storage?
- What else could be done to enhance the data collection, storage and reporting procedure and effectiveness?

**4. Medical Products**

- What medications are available here for the management of SAM children?
- How frequently do you receive medication? Do you receive medication on a timely manner?
- What do you do when you exhaust most of the medications?
- What equipment do you have? What specific equipment have you received for nutrition service delivery?
- Where do you store medications?
- Have you ever received any Nutritional Treatment (NT)/ Nutritional Supplement (NS)/ special food to treat malnutrition? If yes, what have you received and hoe did you use it?

**5. Comments/Suggestions**

- What else could be done for proper screening and treatment of malnourished children?
- Please tell us about problems you encounter regarding absence and job sharing.

**F**. **Government frontline community health workers**

**1. Service Delivery**

- What services are you currently providing through this Community Clinic?
- What services do you provide for maternal and child nutrition?
- What services are available for children with Severe Acute Malnutrition (SAM) and Moderate Acute malnutrition (MAM)?
- What medications/food do you provide for malnourished children?
- What community outreach activities do you conduct? Please explain. (If community outreach activities for SAM and MAM is present)- Where do you refer them?

**2. Work Force**

- How many people work here? What are your job responsibilities?
- What are the training(s) have you received? Have you received any specialized training for identifying severely malnourished children? If yes, what training(s) have you received?
- Please tell us about your daily activities.
- Do you get adequate time to provide nutrition services, i.e. growth monitoring and counseling?
- Who monitors your activities? Does he/she help you to conduct your activities? What else he/she could do to increase your productivity?

**3. Information**

- Have you received any training on how to collect and record data?
- Please tell us how you report service related data?
- Please tell us about problems you encounter regarding data storage?
- What else could be done to enhance the data collection, storage and reporting procedure and effectiveness?

**4. Medical Products**

- What medications are available here?
- How frequently do you receive medication? Do you receive medication on a timely manner?
- What do you do when you exhaust most of the medications?
- What specific equipment have you received for nutrition service delivery?
- Have you ever received any Nutritional Treatment (NT)/ Nutritional Supplement (NS)/ special food to treat malnutrition? If yes, what have you received and how did you use it?

**6. Leadership/Governance**

- Do you have any community group for the community clinic? If yes, please tell us about meetings (frequency), who are the members of the community groups, how decisions are made.

**7. Comments/Suggestions**

- What else could be done for proper screening and treatment of malnourished children?
- Please tell us about problems you encounter regarding absence, job sharing and arranging community meetings?

**G. Community People**

- Please tell us about your community clinic, community group and community clinic Sub-committee members’ community outreach activities.
